# Vinpocetine improved neuropsychiatric and epileptic outcomes in a patient with a *GABRA1* loss‐of‐function variant

**DOI:** 10.1002/acn3.51838

**Published:** 2023-07-11

**Authors:** Cathrine E. Gjerulfsen, Tomasz S. Mieszczanek, Katrine M. Johannesen, Vivian W. Y. Liao, Mary Chebib, Helene A. J. Nørby, Elena Gardella, Guido Rubboli, Philip Ahring, Rikke S. Møller

**Affiliations:** ^1^ Department of Epilepsy Genetics and Personalized Medicine Danish Epilepsy Centre Dianalund Denmark; ^2^ Department of Child Neurology Danish Epilepsy Centre Dianalund Denmark; ^3^ Department of Genetics University Hospital of Copenhagen, Rigshospitalet Copenhagen Denmark; ^4^ Brain and Mind Centre, Sydney Pharmacy School, Faculty of Medicine and Health The University of Sydney Sydney New South Wales Australia; ^5^ Department of Neuropsychology Danish Epilepsy Centre Dianalund Denmark; ^6^ Department of Regional Health Research, Faculty of Health Sciences University of Southern Denmark Odense Denmark; ^7^ Institute of Clinical Medicine University of Copenhagen Copenhagen Denmark

## Abstract

Vinpocetine is a synthetic derivative of the alkaloid vincamine and has been used as a dietary supplement for decades. Following a positive report of the use of vinpocetine in a patient with a loss‐of‐function *GABRB3* variant, we here describe another patient with a loss‐of‐function *GABRA1* variant (p.(Arg112Gln)) who benefited from vinpocetine treatment. This patient was diagnosed with autism spectrum disorder, psychiatric complications, and therapy‐resistant focal epilepsy. Upon add‐on treatment with 40 mg vinpocetine daily for 16 months, the patient experienced an overall improved quality of life as well as seizure freedom. Our findings corroborate that vinpocetine can attenuate epilepsy‐associated behavioral issues in patients with loss‐of‐function GABA_A_ receptor gene variants.

## Introduction

Variants in genes encoding subunits that form the major α1β3γ2 GABA_A_ receptor, *GABRA1, GABRB3*, and *GABRG2*, have been identified to cause different types of epilepsy ranging from mild genetic generalized epilepsy to severe developmental and epileptic encephalopathies.^1^

Recently, Billakota et al.^2^ reported that vinpocetine (Ethyl (3α,16α)‐Eburnamenine‐14‐Carboxylate), a synthetic derivative of the alkaloid vincamine from the periwinkle plant, *Vinca minor*, improved clinical outcome of a 29‐year‐old woman with Lennox–Gastaut syndrome caused by a loss‐of‐function (LOF) variant in *GABRB3*. Upon add‐on treatment with 20 mg vinpocetine three times daily over a period of 9 months, this patient displayed improved language abilities, behavior, and reduced frequency of spike–wave discharge on electroencephalogram (EEG).

In our clinic, we have been treating a 17‐year‐old male with de novo LOF variant in *GABRA1*, who has a history of intractable focal epilepsy and social behavioral disorders. Given the previous success in treating the *GABRB3* variant patient and the fact that vinpocetine is generally considered safe for human consumption, we trialed add‐on treatment with vinpocetine in our patient. A remarkable positive outcome was observed, including reduced seizure frequency and better cognition and behavior.

## Clinical Description

The patient is a 17‐year‐old male born at term by healthy non‐consanguineous parents. Early development was unremarkable; however, mild language delay was noticed at the age of 3 years. He suffered from childhood‐onset fluency disorder that required support from a speech therapist. At the age of 11 months, he presented with his first febrile seizure (FS). In the following 22 months, he experienced five additional bilateral tonic–clonic seizures, provoked by fever (38–38.5°C). Since the third year of life, the patient started to suffer from afebrile focal seizures characterized by, (1) impaired awareness, occasionally evolving to bilateral TC seizure, or (2) impaired awareness and myoclonic jerks in the neck, shoulder, and arm, typically on the left side.

MRI was normal. EEG revealed occipito‐central 2–4 Hz spikes/polyspikes and slow wave complexes accentuated by sleep with anterior spread. Lamotrigine was initiated and he became seizure‐free. At the age of 6 years and 6 months, lamotrigine was replaced with valproic acid (VPA) because of possible adverse effect (vocal tics). In the seventh year of life, attention deficit hyperactivity disorder (ADHD) was diagnosed, and methylphenidate was introduced. He was seizure‐free until the age of 10 years and 11 months, where he started having monthly generalized tonic–clonic (GTC) seizures and daily FS characterized by: (i) hiccups possibly related to myoclonic jerks of the diaphragm, or (ii) eyes flickering and amaurosis, or (iii) visual aura followed by impaired awareness. VPA increase was associated with severe aggressive behavior. Thus, several drugs (eslicarbazepine, lacosamide, zonisamide) were tried without improvement on seizure frequency; in addition, he started to suffer from anxiety and depression. EEG was unchanged, besides the appearance of photosensitivity. At the age of 13 years, he was diagnosed with obsessive compulsive disorder (OCD), autism spectrum disorder, and mild intellectual disability (IQ score at 53). At 15 years old, implantation of vagus nerve stimulator (VNS) in combination with brivaracetam and VPA lead to a reduction of GTC seizures from monthly to only two GTC seizures a year (Table 1). FS persisted weekly, and anxiety and depressive symptoms did not improve. At this age, whole exome sequencing revealed a de novo missense variant in *GABRA1* c.335G>A p.(Arg112Gln), which is a recurrent *GABRA1* variant that has previously been reported in 12 individuals with neurodevelopmental disorders and epilepsy.^1^, ^3^, ^4^, ^5^, ^6^, ^7^, ^8^, ^9^

**Table 1:** Clinical information of EEGs, seizure frequency, behavior problems, administrated medicine, plasma‐values of antiepileptic drugs, and IQ measured by neuropsychological test.

	Before VNS	Before vinpocetine and after VNS	6 months after vinpocetine	12 months after vinpocetine
Interictal EEG	Normal background activity Spikes/polyspikes/spike and slow waves synchronously and asynchronously with alternating side predominance	Normal background activity Spikes/polyspikes/spike and slow waves synchronously and asynchronously spreading from the occipito‐parieto‐posttemporal regions without dominant side	Normal background activity Spikes/polyspikes and slow waves in the occipito‐perieto‐posttemporal regions bilaterally synchronously and asynchronously with alternating side predominance	Normal background activity Spikes/polyspikes and slow waves in the occipito‐perieto‐posttemporalregions bilaterally synchronously and asynchronously with alternating side predominance
Seizure frequency	Type 1, 2, 3, and 4: weekly *(Type 1 lasting up to 30 min)* Type 5: monthly	Type 1, 3, and 4: weekly Type 2: monthly Type 5: only two recorded	Type 1, 2, 3, and 4: none Type 5: only two seizures short after VNP was introduced	Type 1, 2, 3, and 4: none Type 5: none
Behavior	Severe OCD Severe anxiety Depression Aggressive behavior ADHD Infantile autism	Severe OCD Severe anxiety Depression Aggressive behavior ADHD Infantile autism	Mild OCD Mild anxiety Infantile autism	Mild anxiety Infantile autism
Medicine	Sertraline 100 mg/day Aripiprazol Risperidone Valproat 300 mg/day Brivaracetam 260 mg/day	Sertraline 100 mg/day Valproat 300 mg/day Brivaracetam 235 mg/day	Sertraline 100 mg/day Valproat 450 mg/day Brivaracetam 235 mg/day	Sertraline 100 mg/day Valproat 450 mg/day Brivaracetam 235 mg/day
Plasma‐values AED	VPA 257 μmol/L (15 May 2020) BRV 5.3 μmol/L	VPA 169 μmol/L (04 March 2021) BRV 3.7 μmol/L	VPA 220 μmol/L (01 February 2022) BRV 3.0 μmol/L	VPA 160 μmol/L (16 September 2022) BRV 3.5 μmol/L
IQ	53	73	Test not performed	80
Seizure types: Focal seizures consisting of eyes flickering and amaurosis Focal seizures with visual aura followed by impaired awareness Myoclonic movements in upper extremities and head turning left Focal seizures with jerks of the diaphragm (hiccups) Generalized tonic–clonic seizures				

## Treatment with Vinpocetine

Thirteen months after VNS implantation, the introduction of a magistral preparation of vinpocetine 20 mg three times daily was associated with a further reduction of FS, and the patient has currently been seizure‐free for 16 months (Table 1). Repeated EEG showed a gradual decrease of posteriorly predominant epileptic abnormalities. In addition, the patient experienced a remarkable improvement in OCD, anxiety, and depression as well as better scores on neuropsychological tests (Table 1). After 9 months, the vinpocetine dose was reduced to 20 mg two times daily due to recurrent headaches, which then ceased. Periodic assessments of ECG, blood pressure, and blood tests did not show any abnormalities. No adverse drug reactions were reported 16 months after vinpocetine was introduced.

## Functional Analysis

**Figure 1:**
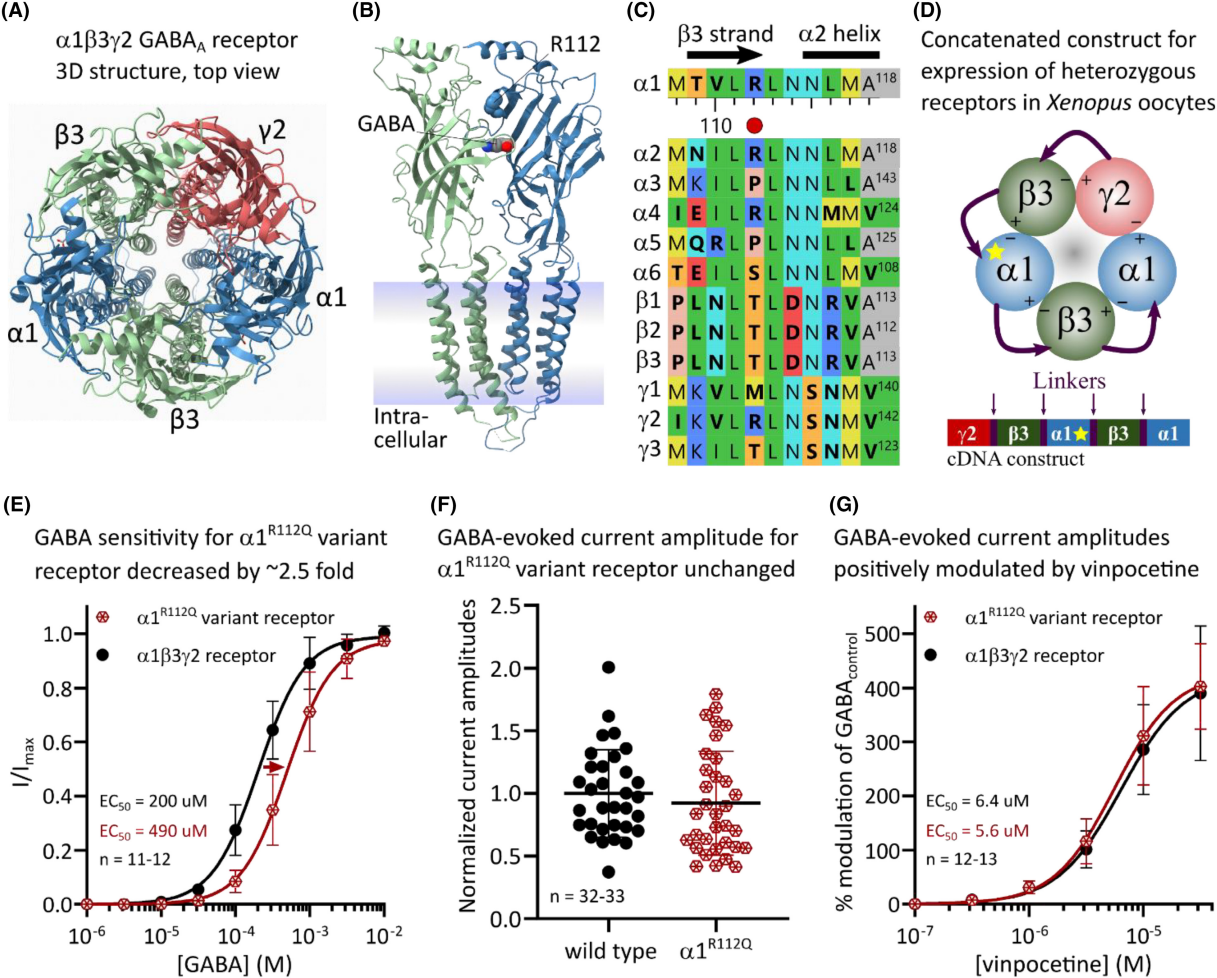
Functional analysis of the Arg112Gln *GABRA1* variant. (A) Top‐view structure of the α1β3γ2 GABA_A_ receptor (pdb:6hup) visualized using UCSF ChimeraX.^20^ (B) Side‐view of a single β3‐α1 interface where the R112 amino acid resides above the binding site for GABA. (C) Sequence alignment of the human α1 subunit with that of other synaptic human GABA_A_ receptor subunits. Only sequences in the immediate vicinity of the R112 position are shown. Alignment was performed using MegAlign Pro 17 and is presented using the shapely color scheme. (D) The concatenated pentameric γ2‐β3‐α1^R112Q^‐β3‐α1 cDNA construct is illustrated with four linkers (purple) and resulting expressed fusion protein viewed from the extracellular side. A yellow star indicates the α1 subunit position that contains the R112Q variant. (E) *Xenopus laevis* oocytes were injected with cRNA and subjected to two electrode voltage‐clamp electrophysiology as previously described.^7^ Normalized GABA‐evoked peak current amplitudes are depicted as mean ± SD as a function of the GABA concentration for *n* = 11–12 experiments for the indicated receptors. A Hill equation was fitted to the data using nonlinear regression (wild type receptor, pEC_50_ = 3.68 ± 0.16, Hillslope = 1.44 ± 0.19, *n* = 12; and α1^R112Q^ variant receptor, pEC_50_ = 3.28 ± 0.19, Hillslope = 1.48 ± 0.28, *n* = 11; where *p* = −Log). The difference in pEC_50_ values is −0.39 ± 0.17, which corresponds to a 2.5‐fold decrease in GABA sensitivity for the variant receptor (*p* < 0.0001, Unpaired two‐tailed *t*‐test). (F) GABA_max_‐evoked peak‐current amplitudes are depicted for *n* = 32–33 experiments. Line with error bars represent mean ± SD. Datasets were not significantly different between variant and the wild type receptor (*p* = 0.2265, Mann–Whitney two‐tailed test). (G) Vinpocetine modulation of GABA‐evoked currents was evaluated by co‐applying a GABA concentration corresponding to EC_7_ with increasing concentrations of vinpocetine. Average modulatory responses ([I_vinpocetine_‐I_control_]/I_control_) are depicted in percent ± SD as a function of the vinpocetine concentration for *n* = 12–13 experiments. A Hill equation was fitted to the data using nonlinear regression (wild type receptor, Top = 420 ± 30%, pEC_50_ = 5.20 ± 0.07, Hillslope = 1.56 ± 0.28, *n* = 13; and α1^R112Q^ variant receptor, Top = 430 ± 20%, pEC_50_ = 5.25 ± 0.05, Hillslope = 1.63 ± 0.24, *n* = 12; where *p* = −Log).

The Arg112 residue is located in the beta‐strand three of the α1 subunit (Fig. 1A–C). The position of this specific amino acid is not particularly conserved among human GABA_A_R subunits including the six known alpha subunits (Fig. 1C). While the Arg112 residue does not directly interact with GABA binding, it is speculated to aid in stabilizing the activated state upon GABA binding.^10^ Electrophysiological experiments were performed to investigate the functional effects of the p.(Arg112Gln) (α1^R112Q^) variant. Patients with de novo *GABRA1* variants are heterozygous for the variant. As pentameric GABA_A_ receptors contain two α1 subunits, patients have mixed receptor populations where each receptor contains either zero (wild type), one (heterozygous), or two (homozygous) variant α1^R112Q^ subunits. As previously shown, receptors with two variant subunits can be expected to be the more severely affected, while receptors with a single variant subunit display less impairment.^11^ As single variant receptors constitute the majority (50%), they are the most important to investigate functionally. Examination of receptors heterozygous for the α1^R112Q^ subunit was accomplished using concatenated receptor constructs as previously described (Fig. 1D).^12^

Electrophysiological analysis revealed that receptors containing the α1^R112Q^ subunit were approximately 2.5‐fold less sensitive to GABA but displayed no change in maximal GABA‐evoked current amplitudes when compared with wild type receptors (Fig. 1E,F). This significant change in GABA sensitivity demonstrates that receptors with a single variant α1^R112Q^ subunit display LOF traits, which is in overall agreement with previous studies of receptors with two variant α1^R112Q^ subunits.^7^ Billakota et al.^2^ reported that vinpocetine positively modulates GABA‐evoked currents, hence this was next investigated. Co‐applications of increasing concentrations of vinpocetine with a fixed concentration of GABA caused concentration‐dependent increases in GABA‐evoked current amplitudes at wild type as well as α1^R112Q^ containing receptors (Fig. 1G). A maximal fitted efficacy of ~400% with accompanying potency of ~6 μM was observed in both cases. Finally, no effects were observed upon applications of vinpocetine alone (up to concentrations of 10 μM). These data suggest that vinpocetine can be considered a positive allosteric modulator of α1β3γ2 receptors and that the Arg112Gln variant does not influence the actions of vinpocetine.

## Discussion

The efficacy of vinpocetine on epileptic seizures has previously been investigated. Meador et al.^13^ treated eight patients with focal epilepsy with 20 mg vinpocetine three times daily without observing significant seizure reduction or any significant side effects. In contrast, Dutov et al.^14^ and Garza‐Morales et al.^15^ reported a considerable decrease in seizure frequency with add‐on vinpocetine treatment in patients with focal epilepsy. While these are obviously contrasting data, it is important to note that the underlying cause for the patient's epilepsy was not disclosed in these studies and it can therefore not be excluded that vinpocetine is beneficial in specific patient populations.

In the recent study by Billakota et al., the cause of the patient's epilepsy was known to be a LOF variant in the *GABRB3* gene.^2^ This patient was diagnosed with Lennox–Gastaut syndrome and add‐on treatment with vinpocetine resulted in a sustained dose‐dependent reduction in spike–wave discharge frequency on EEG as well as an improvement in global impression. Our patient likewise has a LOF variant in a GABA_A_ receptor gene and became seizure free and experienced remarkable improvement in cognition and adaptive skills by adjuvant treatment of 40–60 mg daily vinpocetine. We acknowledge that VNS effects can increase over time; however, the time correlation between the introduction of vinpocetine and the seizure reduction as well as cognitive improvements suggest that these changes were likely related.

Vinpocetine has been shown to have extensive pharmacological actions. It has primarily been thought to inhibit calcium/calmodulin‐dependent cGMP‐PDE^16^ and blocking voltage‐gated calcium as well as sodium channels.^17^ Recently, it was reported that vinpocetine may also potentiate GABA_A_ receptors, and our data supports these findings.^2^ Vinpocetine displayed substantial positive modulation of α1β3γ2 receptors on par with observations for hypnotics and benzodiazepines.^18^ With an observed functional potency of ~6 μM, vinpocetine is more potent at GABA_A_ receptors than it is on most other reported targets (IC_50_ values of 10–50 μM).^19^ Meador et al.^13^ observed blood levels of vinpocetine of ~15 ng/ml in humans following peroral doses of 20 mg three times daily. This corresponds to a concentration of ~40 nM, which appears low in comparison with the observed functional potencies of vinpocetine. However, this observation reflects a single time point and does not reveal the concentrations of vinpocetine available for receptor binding in the human brain. Furthermore, it cannot be excluded that some of the actions of vinpocetine are due to its main metabolite apovincaminic acid which was found in 20‐fold higher concentrations.^13^

Overall, we believe that it is reasonable to speculate that the potentiation of GABAergic activity constitutes an important component for the effects observed in patients with the *GABRB3* and *GABRA1* LOF variants. However, if enhancing the GABAergic system is responsible for the effects of vinpocetine, then caution is required when administering vinpocetine to other patients with *GABR* variants as these can also result in gain‐of‐function variants. Absalom et al.^12^ recently reported adverse reactions to the GABA‐potentiating drug vigabatrin in patients with *GABRB3* GOF variants and a similar scenario could occur with vinpocetine as add‐on treatment in these and other patients that have a GABA_A_ GOF variant.

In conclusion, our findings suggest that vinpocetine may be effective in treating seizure and cognitive behavioral disturbances in patients with LOF GABA_A_R variants and most importantly improve the patients' quality of life. Further studies in larger cohorts including younger patients and with a longer follow‐up are needed to confirm these results and to determine the best dosing practice, long‐term effects, and safety profile of vinpocetine.

## Author Contributions

C.E G., P K A, R.S.M were involved in the conception and design of the study. CEG, TSM, KMJ, VWYL, MC, HAJN, EG, GR, PKA, and RSM were involved in acquisition and analysis of data. C.E G., P K A and R.S.M were involved in drafting a significant portion of the manuscript.

## Conflict of Interest

The authors have no conflict of interest to disclose.

## Funding Information

The Australian National Health & Medical Research Council grant APP1185122 (PKA, VWYL, MC) and the Novo Nordisk foundation grant NNF 0058749 (RSM).
